# Infrapatellar fat pad-derived MSC response to inflammation and fibrosis induces an immunomodulatory phenotype involving CD10-mediated Substance P degradation

**DOI:** 10.1038/s41598-019-47391-2

**Published:** 2019-07-26

**Authors:** Dimitrios Kouroupis, Annie C. Bowles, Melissa A. Willman, Carlotta Perucca Orfei, Alessandra Colombini, Thomas M. Best, Lee D. Kaplan, Diego Correa

**Affiliations:** 1 Department of Orthopaedics, UHealth Sports Medicine Institute, University of Miami, Miller School of Medicine, Miami, FL USA; 2 Diabetes Research Institute & Cell Transplant Center, University of Miami, Miller School of Medicine, Miami, FL USA; 3 Department of Biomedical Engineering, University of Miami College of Engineering, Miami, FL USA; 4 IRCCS Istituto Ortopedico Galeazzi, Laboratorio di Biotecnologie Applicate all’Ortopedia, Milan, Italy

**Keywords:** Mesenchymal stem cells, Regeneration, Acute inflammation, Acute inflammation

## Abstract

The infrapatellar fat pad (IFP) serves as a reservoir of Mesenchymal Stem Cells (MSC), and with adjacent synovium plays key roles in joint disease including the production of Substance P (SP) affecting local inflammatory responses and transmitting nociceptive signals. Here, we interrogate human IFP-derived MSC (IFP-MSC) reaction to inflammatory and pro-fibrotic environments (cell priming by TNFα/IFNγ and TNFα/IFNγ/CTGF exposure respectively), compared with bone marrow-derived MSC (BM-MSC). Naïve IFP-MSC exhibit increased clonogenicity and chondrogenic potential compared with BM-MSC. Primed cells experienced dramatic phenotypic changes, including a sharp increase in CD10, upregulation of key immunomodulatory transcripts, and secreted growth factors/cytokines affecting key pathways (IL-10, TNF-α, MAPK, Ras and PI3K-Akt). Naïve, and more so primed MSC (both) induced SP degradation *in vitro*, reproduced with their supernatants and abrogated with thiorphan, a CD10 inhibitor. These findings were reproduced *in vivo* in a rat model of acute synovitis, where transiently engrafted human IFP-MSC induced local SP reduction. Functionally, primed IFP-MSC demonstrated sustained antagonism of activated human peripheral blood mononuclear cells (PBMC) proliferation, significantly outperforming a declining dose-dependent effect with naïve cohorts. Collectively, our *in vitro* and *in vivo* data supports cell priming as a way to enhance the immunoregulatory properties of IFP-MSC, which selectively engraft in areas of active synovitis/IFP fibrosis inducing SP degradation, resulting in a cell-based product alternative to BM-MSC to potentially treat degenerative/inflammatory joint diseases.

## Introduction

Inflammation of the synovium and infrapatellar fat pad (IFP) characterise progressive Osteoarthritis (OA), a chronic disease that induce articular cartilage (AC) loss often leading to joint replacement. A tight molecular interplay between synovium and the adjacent IFP has been shown to be key during the onset and progression of OA^1–3^. Accordingly, these structures serve as sites of immune cell infiltration and origin of pro-inflammatory and destructive molecules such as tumour necrosis factor-alpha (TNF-α), interferon-gamma (IFN-γ), interleukin 1-beta (IL-1β) and various adipokines, ultimately promoting catabolic responses in AC^4–6^. In advanced stages of OA, additional inflammatory/pro-fibrotic modulators such as connective tissue growth factor (CTGF) in concert with transforming growth factor-beta (TGF-β) induce synovial and IFP fibrosis^7^, further inducing AC damage^4,8–11^.

Mesenchymal Stem Cell (MSC)-based therapy has gained attention as a potential therapeutic alternative for OA given their immunomodulatory and trophic effects^12–14^. MSC have the ability to adopt antagonizing phenotypes, directed by the environment they sense^15,16^. MSC exposed to inflammatory surroundings exhibit immunomodulatory/anti-inflammatory and anti-fibrotic effects, exerted in part through cell-cell contact, the release of paracrine mediators and the enzymatic activity of inducible indoleamine 2,3-dioxygenase (IDO)^17,18^. These molecular mechanisms are enhanced with prior exposure (*i*.*e*. cell priming/licensing) to immune cells and/or environments rich in IFNγ, TNFα and IL-1α/ß^19–21^.

Substance P (SP), a compound secreted by sensory nerve fibres in the synovium and IFP and associated with nociceptive pathways, is also a key modulator of local inflammatory/immune and fibrotic responses. SP-secreting sensory nerve fibres predominate over sympathetic ones in anterior knee pain^22–24^, and it is increased in synovial fluid during joint inflammation^25^. SP effects on immune responses include modulation of cell proliferation, activation and migration to sites of inflammation, and the expression of recruiting chemokines and adhesion molecules^25,26^. SP receptor, neurokinin 1 (NK1R), is widely expressed within synoviocytes, where it regulates the expressions of AC-degrading matrix metalloproteinases (MMPs) and their tissue inhibitors (TIMPs). Furthermore, SP has been shown to enhance the immunomodulatory potential and paracrine activity of MSC^27,28^.

CD10 (also known as neprilysin) is a surface neutral endopeptidase expressed in multiple cell types including the immune system and MSC^29,30^, with enzymatic activity neutralizing various signalling substrates including SP, bradykinin, atrial natriuretic peptide and endothelins^31,32^. For instance, in CD10 KO mice and silenced fibroblasts, exceedingly higher concentrations of SP have been found suggesting its enhanced bioavailability in the absence of CD10^32^. Consequently, as a zinc-dependent metalloprotease, CD10 is capable of terminating inflammatory processes by directly degrading molecular mediators and ligands^32^. However, a direct relationship between CD10 and SP in the context of synovium and IFP inflammation, and more so a potential involvement of resident or injected MSC as a source of CD10 has not been established to date.

IFP constitutes a promising alternative source of MSC to other adult/foetal tissues such as the “standard” bone marrow (BM), given its anatomical relationship with intra-articular structures and its pivotal role in joint homeostasis and disease^33^. Therefore, we assessed phenotypic, transcriptional, secretory and functional responses of IFP-MSC (compared to BM-MSC) to inflammatory (TNFα/IFNγ; TI) and pro-fibrotic (TNFα/IFNγ/CTGF; TIC) priming environments, such as the ones present in synovitis and IFP inflammation/fibrosis. This comprehensive *in vitro* interrogation helps to both understand how these cells may react *in vivo* to harmful environments, as well as to test whether cell priming has inductive effects that could be harnessed in clinical protocols.

## Results

### IFP-MSC growth kinetics, clonogenicity, chondrogenic potential and basal immunophenotypic profile

**Figure 1:**
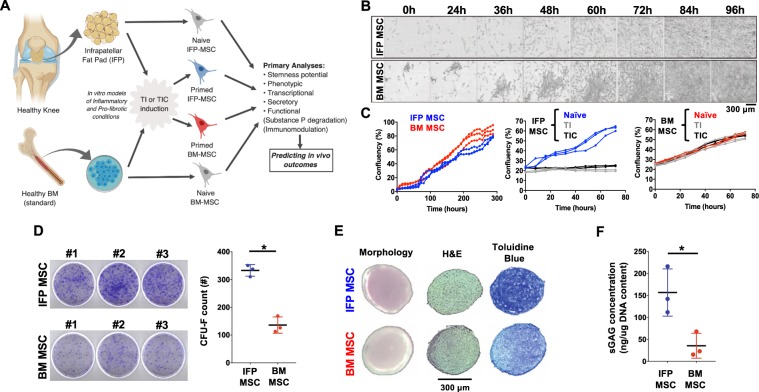
General experimental design and IFP-MSC/BM-MSC growth kinetics, clonogenicity, immunophenotype and chondrogenic capacity. (**A**) General scheme depicting cell sources, stimulation and outcomes (created with BioRender). (**B**) Growth rate time course for representative individual CFU-Fs within IFP- and BM-MSC cultures showing colony confluency. (**C**) Entire culture growth kinetics of naïve IFP- and BM-MSC cultures until confluency (left panel), show comparable growth. Effect of TNFα/IFNγ (TI) or TNFα/IFNγ/CTGF (TIC) priming on IFP- and BM-MSC culture growth kinetics (middle and right panels). Both TI- and TIC- priming resulted in diminished proliferation for IFP-MSC. (**D**) Clonogenic capacity of naïve IFP- and BM-MSC cultures (CFU-Fs per 10^3^ MSC seeded). IFP-MSC generated significantly higher number of CFU-F compared to BM-MSC. (**E**) Chondrogenic differentiation capacity of naïve IFP- and BM-MSC. Chondro-pellet morphology (left photomicrographs), Hematoxylin and Eosin staining (H&E, middle photomicrographs), Toluidine blue staining (right photomicrographs). (**F**) sGAG production on day 21 of differentiation. IFP-MSC showed significantly higher sGAG production compared to BM-MSC. sGAGs production was normalized to total DNA content, comparable between IFP- and BM-MSC. All experiments (n = 3) were performed independently (3 different donors) and data are presented as scatter plots with mean ± SD. Unpaired *t*-test was used for statistical analysis *p < 0.05.

Figure 1A shows the experimental set-up of the study. IFP-MSC demonstrated a slightly slower growth rate beyond 60% confluency compared to BM-MSC (Fig. 1B,C). However, high IFP-MSC clonogenic capacity was evident by significantly (p < 0.005) higher colony-forming unit fibroblasts (CFU-F) numbers compared to BM-MSC (333 ± 21 vs 136 ± 29 CFU-Fs, Fig. 1D).

Chondrogenic differentiation capacity was significantly higher (p < 0.001) in IFP-MSC compared to BM-MSC, evidenced by histological analysis (Toluidine blue) (Fig. 1E), and sulphated glycosaminoglycans (sGAG) quantification, normalized to equal amount of DNA (Fig. 1F).

**Figure 2:**
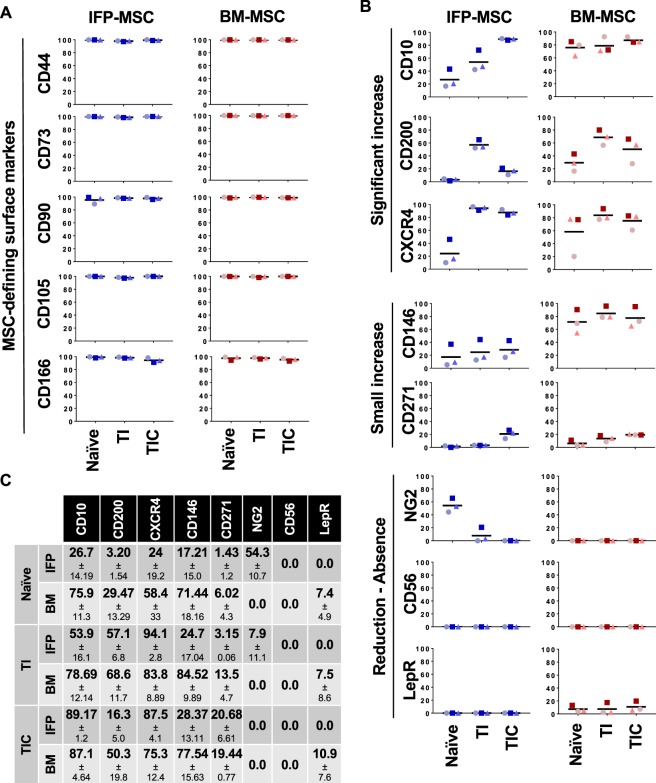
IFP-MSC and BM-MSC immunophenotypic profile pre- and post-TI and TIC priming. MSC-defining (**A**) and multiple (**B**) cell surface markers in pre (naïve) and post TI and TIC priming in both IFP-MSC and BM-MSC, quantified in (**C**). All experiments (n = 3) were performed independently (3 different donors) and data are presented as scatter plots with mean. Individual donors in each MSC type are presented with distinctive shapes and colour tones to allow intra-donor comparisons between naïve and both primed methods.

IFP-MSC showed a similar immunophenotype to BM-MSC for the commonly expressed (>90%) MSC-defining markers (Fig. 2A,C). On the other hand, CD146, CXCR4, CD10 and CD200 were expressed more in BM-MSC than IFP-MSC, while NG2 only in IFP-MSC. CD271, LepR and CD56 had low-to-negative expression in both cell types (Fig. 2B,C).

### Effects of priming on growth kinetics

Growth kinetics of IFP and BM-MSC were evaluated with and without TI or TIC for 72 h. Both priming schemes halted IFP-MSC proliferation at approximately 20% confluency compared to naïve-MSC culture, which reached ~60% confluency (Fig. 1C). In sharp contrast, BM-MCS proliferation was not affected by either scheme reaching in parallel ~55% confluency (Fig. 1C).

### Effects of priming on immunophenotypic and transcriptional profiling

Priming had no effect on the MSC-defining markers (Fig. 2A). CD146 expression was increased in IFP-MSC with both TI and TIC priming, whereas in BM-MSC only TI priming increased expression. CD10, CD200, CXCR4 and to a lesser extent CD271 and CD146 were all increased with both priming schemes, with varying effects and the differences more pronounced in IFP-MSC. TIC had a more dramatic increase in CD10, while TI in CD200 and both equally in CXCR4. NG2, on the other hand, was reduced with both priming schemes in IFP-MSC (Fig. 2B).

**Figure 3:**
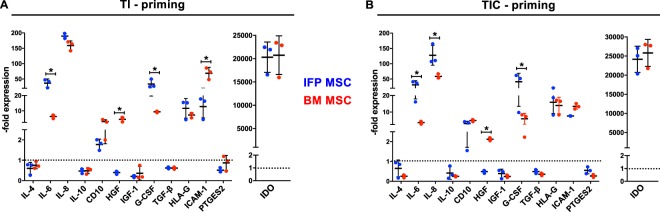
Transcriptional profiling of IFP-MSC and BM-MSC pre- and post-TI and TIC priming. Transcriptional assessment of IFP-MSC and BM-MSC pre and post TI (**A**) and TIC (**B**) priming, showing similar profiles between both methods but with statistical differences (p < 0.05) between MSC types in *HGF*, *IL-6*, *IL-8*, *ICAM-1* and *G-CSF* gene expression (asterisk *). Nevertheless, the trends are similar, including a low *IL-6/IL-8* ratio. All experiments (n = 3) were performed independently (3 different donors) and data are presented as scatter plots with mean ± SD. All shown genes have overall (both MSC) statistically significant (p < 0.05) differences compared with naïve cells (set up to 1 - dotted line - to calculate the fold change). One-way ANOVA for multiple comparisons was used for statistical analysis.

Transcriptional profiles of TI primed IFP and BM-MSC were similar, except *CD10* expression was upregulated more in BM-MSC and *HGF* gene expression was down regulated in IFP-MSC but strongly upregulated in BM-MSC (Fig. 3A). There was a significant (more than 2-fold) up-regulation of *IL-6*, *IL-8*, *IDO*, *HLA-G*, and *G-CSF* gene expression in both TI-primed MSC, with *IDO* gene expression showing the highest up-regulation. *G-CSF* had 50x higher expression in IFP-MSC compared to BM-MSC. Even though IL-6 and IL-8 showed strong upregulation of gene expression in both groups, the *IL-6*/*IL-8* gene expression ratio remained low (<1.0).

Transcriptional profiles of TIC primed MSC (Fig. 3B) were similar to TI-primed. There was significant up-regulation of *IL-6*, *IL-8*, *IDO*, *CD10*, *G-CSF*, *HLA-G*, and *ICAM-1* genes. However, *IL-6*, *IL-8*, and *G-CSF* expression showed an average of 9, 12, and 46 times higher expression whereas ICAM-1 showed in average 3-fold times lower expression in IFP-MSC compared to BM-MSC. Specifically, upon TIC priming *CD10* expression was upregulated in both IFP and BM-MSC. *IDO* expression had greater upregulation in TIC-induced IFP-MSC compared to TI-primed. Similar to TI priming, the average *IL-6*/*IL-8* gene expression ratio remained low (<1.0).

### Effects of priming on the secretory profile

**Figure 4:**
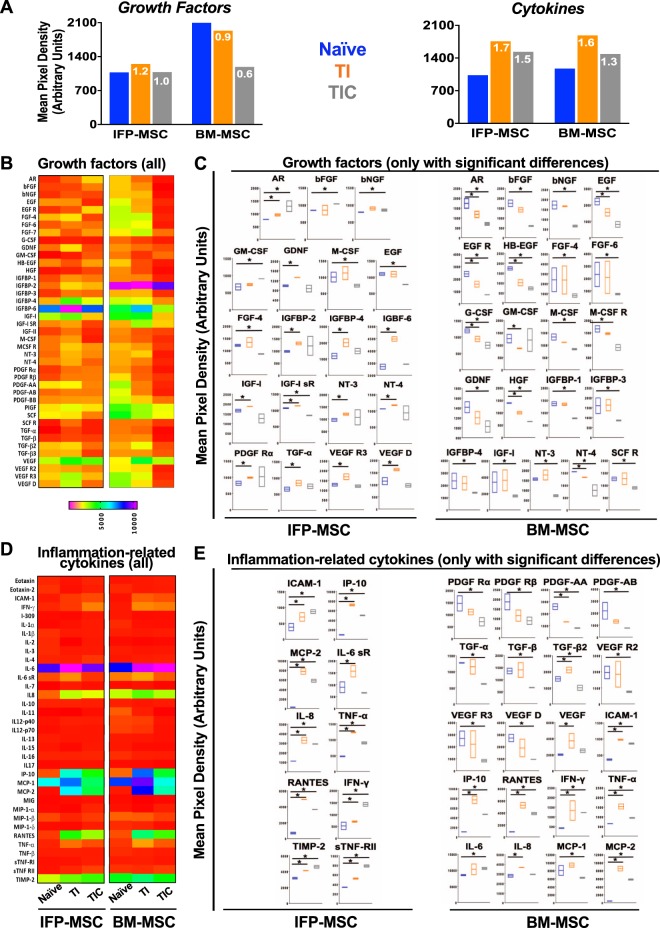
IFP-MSC and BM-MSC secretory profiling pre- and post-TI and TIC priming. (**A**) Growth factors and cytokines secretion in all groups, showing overall quantified fold signal (TI and TIC compared to naïve). (**B**,**D**) Heat maps of the growth factor (**B**) and inflammation-related cytokines (**D**) secretory profile of naïve, TI and TIC primed IFP- and BM-MSCs, showing only proteins with statistically significant changes in C and E, respectively. Heat maps colours are assigned according to a molecule concentration relative scale, from 0 to 10,000. All experiments (n = 2) were performed independently (2 different donors) and data are presented as floating bars with minimum, maximum and mean. Paired and unpaired *t* tests and one-way ANOVA for multiple comparisons were used for statistical analysis *p < 0.05.

Naïve BM-MSC secreted overall higher levels of growth factors compared to IFP-MSC (Fig. 4A), however, only insulin-like growth factor binding protein (IGFBP)-2 and stem cell factor (SCF) showed statistically significant increases (*p* < 0.0001 and *p* < 0.05, respectively). TI-primed IFP-MSC significantly up-regulated the secretion amphiregulin (AR), IGFBP-2, -4, -6, and neurotropins (NT) -3, -4 (*p* < 0.05). In contrast, TI-primed BM-MSC up-regulated secretion of TGF-β2 and VEGF and down-regulated secretion of EGF, G-CSF, HB-EGF, HGF, NT-4 (Fig. 4B,C). TIC primed IFP-MSC up-regulated secretion AR, bFGF, GM-CSF and down-regulated secretion EGF, FGF-4, IGF-IsR, M-CSF, PDGF-AA. TIC-primed BM-MSC significantly (*p* < 0.05) down-regulated the secretion of 31 of the 40 growth factors tested indicating that CTGF addition to the priming cocktail has a dramatic effect in secreted growth factor regulation (Fig. 4B,C).

Both naïve and primed IFP and BM-MSC secreted similar levels of cytokines (Fig. 4D,E). TI primed IFP-MSC significantly up-regulated secretion of ICAM-1, IL-6sR, IP-10, MCP2, RANTES, sTNF-RII, and TIMP-2 (*p < 0*.*05*). In BM-MSC, TI priming resulted in up-regulated secretion of ICAM-1, IP-10, MCP-2, and RANTES. TIC primed IFP-MSC significantly up-regulated the secretion of ICAM-1, IL-8, IP-10, MCP-2, RANTES, TIMP-2, and sTNF-RII (*p < 0*.*05*). TIC primed BM-MSC up-regulated ICAM-1, IL-6, IP-10, MCP-1 and 2, RANTES.

### Protein-protein interactomes and pathway analysis

**Figure 5:**
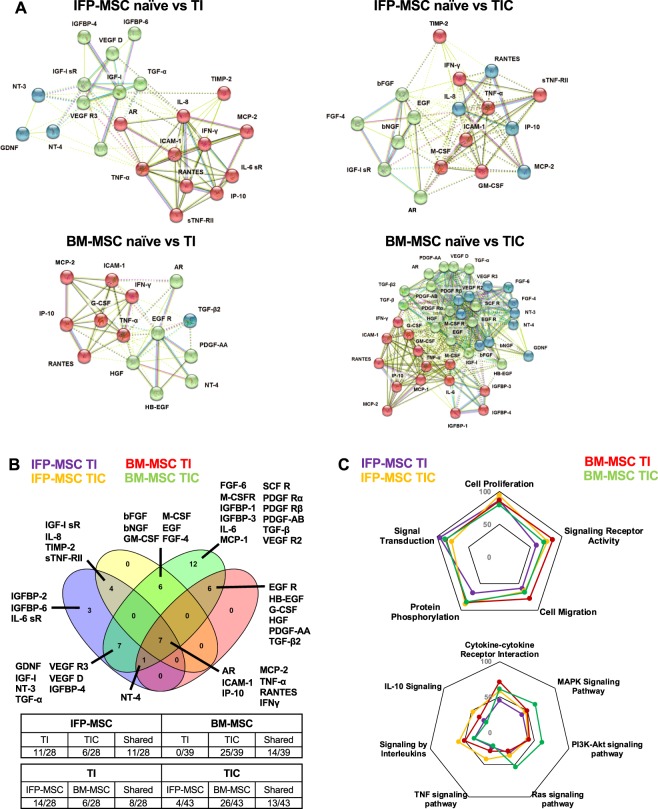
Protein interactomes of IFP-MSC and BM-MSC secretory profiling pre- and post-TI and TIC priming. (**A**) STRING analysis of those proteins with statistical differences between naïve and TI-/TIC- priming conditions (total of 46) was performed using all available interaction sources and 0.4 as a confidence interaction score. K-means algorithm resulted into firm clustering of the differentially expressed factors into 3 groups (different colours) indicating high protein interaction within each group. (**B**) Venn diagram showing shared proteins among the groups, with AR, ICAM-1, IP-10, MCP-2, TNF-α, RANTES and IFN-γ present in both MSC types and in inflammatory and pro-fibrotic conditions. Table shows the number of proteins shared by different groups and conditions. (**C**) Biological processes and KEGG/reactome pathways analyses revealed different type and number of proteins affected post-TI and -TIC priming for both IFP-MSC and BM-MSC. Graph percentages represent the number of proteins involved in a specific pathway/function, related to the total amount of proteins differentially expressed between naïve and TI-/TIC-priming conditions.

To assess the relationship among the proteins identified as significantly (*p* < *0*.*05*) different between naïve and TI-/TIC-primed MSC, protein association network analysis was performed using STRING 11.0 software. In general, all proteins appeared interconnected at least through one association and according to K-means clustering algorithm can be clustered into 3 groups each one representing highly interactive proteins (Fig. 5A). All K-means clustering networks show high protein-protein interaction (PPI) enrichment (p-value < 1.0e-16) and an average local clustering coefficient >0.7 indicating that the proteins used are at least partially biologically connected. Venn diagram analysis indicated that out of the total 46 proteins (growth factors and cytokines) significantly altered, several are shared between different conditions (Fig. 5B). All four MSC groups share significant changes in 7 upregulated proteins (AR, ICAM-1, IP-10, MCP-2, TNF-α, RANTES, IFN-γ) indicating that these molecules are increased in both inflammatory and pro-fibrotic conditions. TI- and TIC-primed groups share 11/28 proteins (39.3%) in IFP-MSC, whereas 14/39 proteins (35.9%) in BM-MSC. From the non-shared proteins, 11 are specific for TI and 6 for TIC in IFP-MSC, while in BM-MSC all remaining 25 proteins are exclusive for TIC, suggesting that in BM-MSC TIC stimulation is a highly specific phenomenon. Comparing MSC types responses, TI generated them to share 8/28 molecules (28.5%), while TIC 13/35 (37.1%), indicating that the majority of the molecular response is cell-specific, beyond a “core” MSC secretory effect.

Five biological processes were evaluated, namely, regulation of cell proliferation (GO:0042127), regulation of signalling receptor activity (GO:0010469), positive regulation of cell migration (GO:0030335), regulation of protein phosphorylation (GO:0001932 and 0050730), and regulation of signal transduction (GO:0009966) (Fig. 5C). TIC priming in IFP-MSC result in higher number of proteins involved in cell proliferation and migration, receptor activity, and protein phosphorylation compared to TI priming. Only proteins involved in regulation of signal transduction show a reduction in numbers compared to TI priming. In contrast in BM-MSC, TIC priming result in decreased number of proteins involved in cell proliferation and migration, and receptor activity compared to TI priming. Additionally, 7 KEGG and reactome pathways were evaluated including cytokine-cytokine receptor interaction (hsa:04060), MAPK signalling (hsa:04010), PI3K-Akt signalling (hsa:04151), Ras signalling (hsa:04014), TNF signalling (hsa:04668), signalling by interleukins (HSA:449147), IL-10 signalling (HSA:6783783) (Fig. 5C). TIC-primed IFP-MSC demonstrated higher number of proteins involved in all the evaluated pathways compared to TI-primed IFP-MSC. In contrast, TIC-primed BM-MSC show higher number of proteins involved only in MAPK, PI3K-Akt, and Ras signalling pathways compared to TI-primed BM-MSC (Supplementary Table S1).

### Substance P *in vitro* immuno-quantification and CD10 immunolocalization

**Figure 6:**
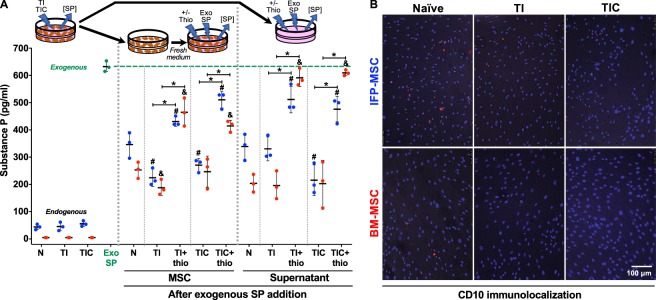
Substance P immuno-quantification. (**A**) ELISA-based quantification of endogenous and exogenously-added SP to naïve (N), TI- and TIC-primed IFP-MSC (blue) and BM-MSC (red) cells and supernatant groups, with or without previous CD10 inhibition with thiorphan (thio). Wells with only exogenously-added SP to medium (Exo SP - green) were used as baseline to assess the degradative effects of cells and supernatants. A diagram represents the source of the samples obtained for the measurements. (**B**) CD10 immunolocalization in naïve, TI and TIC-primed IFP-MSC and BM-MSC. Data from three (n = 3) independent experiments (3 different donors) are presented as scatter plots with mean ± SD. *p < 0.05 between +/− thiorphan for each condition; ^#^p < 0.05 with respect to naïve (N) IFP-MSC or supernatant; ^&^p < 0.05 with respect to naïve (N) BM-MSC or supernatant. Paired and unpaired *t* tests were used for statistical analysis.

Endogenous SP was low in both MSC types (all groups), with no changes after either TI or TIC priming (Fig. 6A). The levels of exogenously-added SP were significantly reduced (p < 0.005) in cells and supernatants in all groups (naïve, TI and TIC-primed), when compared with exogenous SP alone. BM-MSC showed a more pronounced reduction (statistically significant, p < 0.005) than IFP-MSC in naïve cells and supernatants, while comparable in primed groups. These effects on SP were significantly abrogated (p < 0.005) by the CD10 inhibitor thiorphan, more evident in BM-MSC supernatants than in cells as they were similar to exogenous SP (Fig. 6A).

CD10 was identified in MSC as a concentrated punctuate signal around cells (Fig. 6B), highly present in naïve MSC (more pronounced in IFP than BM) and reduced in all primed cells (both types).

### Substance P *in vivo* assessment

**Figure 7:**
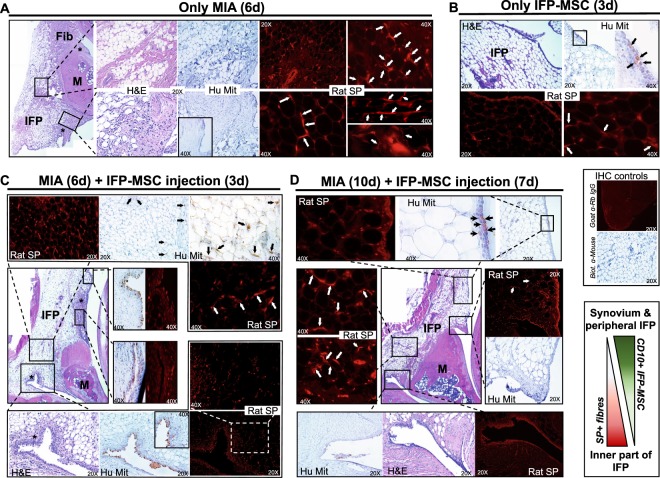
Rat model of acute synovium/IFP inflammation. Hematoxylin and Eosin staining, human mitochondria (Hu Mit) and rat Substance P (Rat SP) immunolocalization in sagitally-sectioned knees of representative rats injected only with MIA (**A**), only with IFP-MSC (**B**), and with both MIA and IFP-MSC (C and D). White arrows help localize the bead chain-like structures of SP-positive nerve fibres between adipocytes inside the IFP as previously described^22,79^. Compared with healthy rats (**B**) where few SP^+^ fibres were observed, MIA-treated rats show increased SP presence both at the periphery and the inner part of the IFP (A). The groups receiving IFP-MSC (**C**,**D**), show a dramatic reduction in SP positivity at the periphery of the IFP (close to the synovium), while less pronounced reduction in the inner part. Black arrows help localize engrafted human IFP-MSC, predominantly in synovium and to a lesser extent the IFP after 3 days of injection (**C**), significantly reduced after 7 days of injection (**D**). Of note, a small amount of IFP-MSC were localized in a synovial spot in healthy rats (**B**), while no signal was detected in rats receiving only MIA with no cells (A), supporting the high specificity of the signal. 4X, 20X and 40X objectives generate magnifications of 40, 200 and 400 times. * areas of synovitis; Fib = areas of fibrosis; M = meniscus; IFP = infrapatellar fat pad.

SP-positive sensory hyperinnervation has been associated with inflammatory conditions in the joint^22–25,34^; therefore, we used a rat model of induced acute synovitis/IFP inflammation to test whether the *in vitro* SP degradation generated by IFP-MSC (in our experiments) could be reproduced *in vivo*. In addition to signs of synovitis and early fibrotic changes of the IFP (Fig. 7A), we confirmed the hyperinnervation by SP-positive nerve fibres 6 days after the intra-articular injection of Mono-iodoacetate (MIA), compared with knees without the inflammatory induction (Fig. 7A,B). Of note, the majority of animals that received IFP-MSC showed an overall reduction in signs of synovitis and especially IFP fibrosis, suggesting that the cells were slowing down and/or antagonizing the progression of the disease. The SP positivity was significantly diminished in rats after 3 days of single intra-articular injection of IFP-MSC, more pronounced in peripheral areas of the IFP (close to the synovium), whereas inner parts showed some remaining SP-positive fibres (Fig. 7C). This pattern of SP absence spatially correlates with presence of engrafted human IFP-MSC, which were immunolocalized through a specific anti-human mitochondria antibody, suggesting that engrafted IFP-MSC indeed are capable of inducing SP degradation in their vicinity. In that regard, Fig. 7C shows predominant synovial engraftment of injected IFP-MSC, with some scattered cells migrated towards the inner part of the IFP. The overall engraftment efficiency was significantly greater compared with knees that received only IFP-MSC without MIA, suggesting that injected IFP-MSC indeed “require” danger signals arising from inflamed tissues to home and engraft. The presence of IFP-MSC was dramatically reduced in knees analysed at day 10 (7 days after IFP-MSC injection), demonstrating that engrafted cells have a short residence time period (Fig. 7D). Nevertheless, the SP reduction, mainly in peripheral regions of the IFP still persists.

### Immunopotency assay

**Figure 8:**
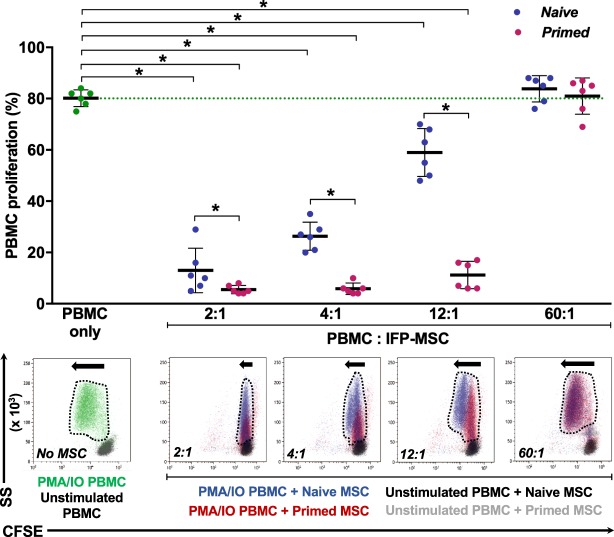
Immunopotency assay (IPA) with human activated PBMC and IFP-MSC in co-culture. The percentage of CFSE-labelled PMA/IO-activated PBMC proliferation (relative to unstimulated PBMC) was measured in PBMC only and in co-cultures with naïve and TI-primed IFP-MSC at various ratios (2:1, 4:1, 12:1, 60:1 PBMC:IFP-MSC). Naïve IFP-MSC (blue) abrogated PBMC proliferation in a declining dose-dependent manner, while TI-primed cells (red) sustainably affected PBMC proliferation even at a 12:1 ratio. At a 60:1 ratio, the interaction with either IFP-MSC slightly augmented PBMC proliferation. PBMC proliferation is quantified (top), whereas corresponding representative flow cytometry overlay data (bottom) shows proliferation magnitude with dotted line shape and arrow sizes. All co-cultures were performed in duplicate, with every MSC donor (n = 3) co-cultured in independent experiments with two different PBMC populations obtained from unrelated donors. Data are presented as scatter plots with mean ± SD, with 6 data points per conditions (3 MSC ×2 PBMC). Paired and unpaired *t* tests and one-way ANOVA for multiple comparisons were used for statistical analysis *p < 0.05.

CFSE-labelled, phorbol myristate acetate and ionomycin (PMA/IO)-activated human PBMC showed a proliferation of ~80%, which was abrogated in a dose-dependent manner by co-culture with naïve IFP-MSC (Fig. 8). Reducing the number of MSC in the co-culture limited their ability to antagonize PBMC proliferation. TI-primed cells, on the other hand, were significantly more effective than naïve cohorts, exhibiting a constant inhibition up to a 12:1 ratio. At a 60:1 ratio, all IFP-MSC groups were unable to affect PBMC proliferation, in fact, generated a slight increase (Fig. 8).

## Discussion

In the present study, we found that cell priming enhances IFP-MSC immunomodulatory properties by modifying phenotypic (*e*.*g*., CD10 enrichment) and secretory profiles, while modulating local key peptides such as SP both *in vitro* and *in vivo* in a rat model of acute synovitis. These types of observations could provide a rationale for further testing of the IFP as a viable source of MSC to manufacture cell-based products for OA cell therapy.

Inflammation and immune events are common features in OA development and progression, with various types of immune cells including monocytes, mast cells, T and B cells infiltrating the synovial membrane and the underlying IFP^35^. These infiltrates, along with IFP and synovial resident inflammatory-type M1 macrophages, produce vast amounts of pro-inflammatory cytokines (*e*.*g*., TNFα, IFNγ and IL-1α/ß), adipokines (*e*.*g*., leptin and adiponectin) and AC catabolic factors (*e*.*g*., MMPs). This is followed by a gradual release of pro-fibrotic mediators (*e*.*g*., CTGF), promoting IFP fibrosis and further joint alterations^8,36^. The resulting tight molecular crosstalk between synovium and IFP supports the notion of IFP as a key player in the pathophysiology of joint disease^2^. TNFα and IFNγ activities as central mediators of inflammation have been extensively documented^37^, thus will not be revisited here. CTGF (CCN2) is a member of the CCN family of molecules, which have been proposed as a new class of inflammation modulators^38^. CTGF has been implicated in the control of joint homeostasis and inflammatory responses^8,11,39–41^, as well as a predictor of OA severity^42^. Beyond its contributions to inflammation, CTGF also has pro-fibrotic effects on synovium and IFP in concert with TGF-β^7,43^.

Based on these activities, in the present study we conducted a phenotypic, transcriptional, secretory and functional characterization of IFP-MSC, comparing their *in vitro* responses to inflammatory and pro-fibrotic conditions with those generated by the current cell-based therapy “standard” BM-MSC. Our results not only provide information on how resident IFP-MSC may sense and respond to inflammatory and pro-fibrotic microenvironments during joint disease progression, but also how exogenously-administered MSC are influenced *in vivo* after injection into a pathological joint. Furthermore, our findings provide critical information on the potential advantages of *ex vivo* priming MSC with inflammatory and/or pro-fibrotic cocktails as a potential way to acquire or enhance specific properties and attributes prior to their clinical use. Overall, cell priming resulted in a phenotypic shift and a significant enhancement of the immunomodulatory properties of both MSC types, more pronounced in IFP-MSC and partially reversed (secretory response) by TIC in BM-MSC. Mechanistically, the CD10-dependent degradation of SP observed *in vitro* with both MSC and *in vivo* with transiently engrafted IFP-MSC to inflamed synovium and IFP, may help explain some of the effects clinically observed with cell-based therapy in OA (*e*.*g*., pain control), while offering additional evidence of how MSC modulate local immune responses.

IFP-MSC demonstrated plastic-adherence, possessed a fibroblast-like morphology, and showed comparable growth rates to BM-MSC under basal conditions. TI and TIC priming halted expansion of IFP-MSC but not BM-MSC. This could indicate an increased cell cycle sensitivity of IFP-MSC to TNFα and IFNγ, which has been reported for other cell types^44^. IFP-MSC sustained high CFU-F formation through passaging, demonstrated increased chondrogenic differentiation, even exhibiting an increased extracellular matrix production compared to BM-MSC (~6 times more sGAG after 21 days of induced differentiation). These results align with previous studies indicating that IFP-MSC are suitable for cartilage cell-based treatments^45^, or could be used to engineer cartilaginous grafts in a clinical setting^46^.

Phenotypically, both naïve and primed IFP- and BM-MSC had similar MSC phenotypic profiles of highly positive (>90%) markers as established by ISCT^47,48^. In accordance with previous reports^49^, the CD146 marker which defines the pericytic origin of BM-MSC^50^, was lowly expressed in all three IFP donors. NG2, another pericytic marker, showed significantly higher expression in IFP-MSC than BM-MSC, indicating that IFP-MSC retain their perivascular immunophenotype despite low CD146 levels. CD271 was low in both MSC, as its expression is heavily impacted by serial passaging^51^. CXCR4 expression, which confers MSC migratory and homing capacities to injured sites^52^, was high in both MSC. CD10 and CD200 were higher in BM-MSC than in IFP-MSC under basal conditions. Those markers have been reported to discriminate subcutaneous and visceral adipose tissue depots based on their specific expression^29^. The distinct nature of IFP compared with subcutaneous adipose tissue, in which CD10 is high, may explain the lower expression observed here. Furthermore, CD10 expression varies depending on donor and culture passage^47^. CD200, a visceral adipose tissue-rich marker^29^, has been implicated in various immunomodulatory activities of myeloid and other immune cells, and is induced in BM-MSC by inflammatory cues^53^. In this study, IFP tissue was obtained from non-arthritic patients undergoing elective arthroscopy in whom active inflammation (*i*.*e*. synovitis) was ruled out by MRI, consistent with the low CD200 expression levels in naïve IFP-MSC. Based on the significant expression differences in CD200 between IFP- and BM-MSC, we propose it as a possible marker for active inflammatory responses that merits further investigation.

In both MSC, priming resulted in significant phenotypic changes, more pronounced in IFP-MSC, interpreted as acquired adaptations to their stimulating conditions. The low expression/absence of CD56, along with increasing CD271 and CD146 suggests a perivascular-enriched phenotype, based on previous reports^50,54^. More importantly, it shifted the cells towards an immunomodulatory phenotype, again more pronounced in IFP-MSC. The most notable changes were observed in the injury response/immunomodulatory-associated markers CD10, CD200 and CXCR4, which were significantly increased with both priming schemes in IFP-MSC and less in BM-MSC. This aligns with previous studies showing that MSC priming with inflammatory cytokines can significantly increase MSC homing to injured tissues and improve their immunomodulatory properties (reviewed in^55^). Notably, TIC differentially affected CD10 and CD200 altering the already positive response to TI alone, as it further increased CD10 while reversed the increment in CD200. This may suggest that the addition of CTGF discriminates further the phenotypic response of IFP-MSC to purely inflammatory and inflammatory/pro-fibrotic environments, arguably related with the stage of the disease.

We observed significant differences in the immunomodulatory secretory profiles of both MSC types upon TI and TIC priming. Of note, TIC induces a highly specific response in BM-MSC, as most of the molecules altered are not changed by TI. On the contrary, IFP-MSC responses were more balanced. The overall “signature” response in all cases involves the upregulation of key immunomodulatory molecules including the monocytes and T leukocyte recruitment chemokines MCP-2 and RANTES and the immunosuppressive proteins AR, ICAM-1, IP-10. AR has been shown to be crucial for the efficient CD4^+^ regulatory T cell (CD4^+^ Treg) function once migrated to sites of inflammation^56^. ICAM-1 expression in MSC relates with an enhanced immunosuppressive capacity, both *in vitro* and *in vivo*^57^, while IP-10 secretion directly correlates with decreased T cell proliferation^58^. Taking into account the transcriptional upregulation of both IL-6 and IL-8 (more pronounced in IL-8), and the selective IL-8 increased protein levels in IFP-MSC, we could speculate that a “beneficial” low IL-6/IL-8 ratio is generated, as previously reported for inflammatory and neoplastic environments^59,60^. Furthermore, this may align with the increased levels found for the IL-6 inhibitor IL-6sR, which along with another elevated cytokine inhibitor (sTNF-RII) have been described as relevant to control the inflammatory process^61^. In terms of biological processes, various categories were highly affected including signal transduction, cell proliferation, protein phosphorylation, signalling receptor activity and cell migration. Combined, these effects empower the cells to respond to inflammation/injury by increasing their number, migrating to active sites of damage and altering key cascades known to affect local immune responses. Regarding the type of signalling pathways affected, a polarization was observed between IFP-MSC and BM-MSC. The former had profound effects mainly in interleukins (especially the immunosuppressive IL-10-dependent pathway) and TNF, whereas the latter more influence on MAPK, PI3k-Akt, and Ras signalling. In our study, TI and more so TIC priming resulted in secretion of proteins involved in all inflammation-related pathways, more evident in the Ras signalling pathways. A major role of CTGF in activating MAPK pathway via Ras and PI3K/Akt signalling has been previously reported through its interaction with the tyrosine kinase receptor 1 (TrkA), controlling cell survival, proliferation and inflammatory responses^62,63^. Even though not evaluated in our study, putatively CTGF/TrkA binding in TIC-primed IFP-MSC may explain the increased interleukins and significant IL-10 signalling involvement compared to TI-primed IFP-MSC.

The pronounced CD10 enrichment in IFP-MSC after both TI and TIC priming, paralleled with the significant reduction in SP levels exercised by naïve but more so by primed cells, suggested to us a CD10-dependent SP degradation. The abrogation of this effect after inhibiting the enzymatic activity of CD10 with thiorphan strongly supports this hypothesis. Furthermore, a general correlation between CD10 expression and SP levels was observed. In that respect, naïve BM-MSC showed a higher CD10 expression and a more marked SP degradation than naïve IFP-MSC (both in cells and supernatants). Once primed, both cells exhibited comparable high CD10 expression levels and similar enhanced SP degradative effects. Supernatants obtained from the same stimulated cell cultures mirrored the SP degrading activities observed with the cells, suggesting that the mechanism(s) used by MSC to degrade SP is(are) not necessarily cell bound, but rather released. Moreover, the similar inhibition observed with thiorphan in supernatants also points to CD10, but now as a released molecule (supernatants were proven to be cell-free). It has been suggested that microvesicles released from MSC may recapitulate some of the functions of their parent cells^64–66^. In that regard, adipose-derived MSC secrete exosome-type of microvesicles with enzymatically active CD10^67^. Therefore, we can speculate that IFP- and BM-MSC secrete CD10-bound exosomes, which once in the supernatant degrade surrounding exogenously-added SP. Functionally, the higher “retention” of CD10 observed in naïve IFP-MSC cultures after SP addition, along with a limited SP degradation may support the concept of CD10 release as required for its degradative activity. This may be further supported by the enhanced inhibition of SP degradation by thiorphan observed in supernatants, compared with their parent cells (especially in BM-MSC).

The CD10-dependent SP degradation effect may help to keep local levels of SP within the synovium/IFP under control. This is relevant given the multifaceted functions of SP in homeostasis as well as in pathological states within the joint^25,26,34,68,69^. We confirmed the i*n vitro* SP degradation in our rat model of acute synovitis/IFP inflammation, in which the injection of MIA rapidly triggers an inflammatory response in those structures, ultimately leading to fibrotic changes^70^. First of all, we found neither clinical nor histological signs of xeno-rejection of the human material by the host. This supports the notion of the immunoevasive properties of MSC^71^. A striking spatial inverse correlation was found between IFP-MSC presence and absence of SP^+^ fibres, especially at the synovium and the periphery of the IFP. We and others have previously shown engraftment of MSC to injured tissues (*e*.*g*., bone marrow^72^). However, to the best of our knowledge there is no previous report showing this degree of specificity of injected MSC to homing and transiently engrafting (<7d) to inflamed synovium, beyond other reports showing meniscus and AC engraftment for longer time periods^73^. The discrepancies observed with the study by Li *et al*. may relate to the different models applied. While Li *et al*. used a mechanical model of OA (partial meniscectomy), which explains the partial meniscal engraftment, we used a rapid chemically-driven synovitis. The selective engraftment to areas of active synovitis reinforces the idea of MSC sensing and following injury signals such as TNFα, IFNγ and CTGF, the ones we used for priming our cells *in vitro*. In addition to transient synovial engraftment, we observed migration of cells towards the inner part of the IFP, although in small numbers. Again, a spatial correlation can be made between presence of cells and SP^+^ fibres, now at the inner part of the IFP, where few cells are present in areas of SP^+^ hyperinnervation. This may be secondary to an inefficient penetration of MSC to those locations, in part due to an initial reduced cell dose administered (10^5^ cells). Current efforts are centered on administering varying amounts of cells to evaluate a possible uniform penetration into the inflamed/fibrosing IFP.

The immunomodulatory potential of IFP-MSC has been previously reported, specifically related with their negative influence on PBMC proliferation^74^. Here, we confirmed that and further determined an underlying declining dose-dependent effect exerted by naïve IFP-MSC. To the best of our knowledge, this is the first time that such a dose-dependent effect is reported, certainly in IFP-MSC. Both naïve and TI-primed IFP-MSC comparably mitigated activated PBMC proliferation at a high dose (low PBMC:MSC), an influence that is remarkably dissipated in naïve IFP-MSC by reducing their numbers by half or more (4:1 and further ratios), but not in TI-primed IFP-MSC which remain effective up to a 12:1 ratio. At an outstanding low MSC dose (60:1 ratio) both naïve and TI-primed were equally ineffective. These findings have significant clinical implications, as they would support the use of fewer numbers of MSC as part of cell-based therapy protocols, as long as they are primed a priori. These are the directions of current research efforts.

The strong immunomodulatory attributes of IFP-MSC could be explained by the acquisition/enrichment of a strong functional phenotype discriminated by high CD10, CD200 and CXCR4, an enhanced immunosuppressive secretome, upregulated pathways such as IL-10, and upregulated key immunomodulatory molecules including IDO, HLA-G and the cytokine inhibitors IL6-sR and sTNF-RII. We hypothesize that the degradation of SP, enhanced in primed MSC and supernatants, also plays a contributing role based on the known effects of SP regulating immune responses including T cell proliferation via IL-2, Th1 shift and overall inflammatory cytokine production^25^. Based on the established anti-inflammatory effects of blocking SP signalling effect in multiple diseases (reviewed in^25^), further studies are needed to potentially establish the MSC-derived CD10-dependent SP degradation mechanism as a viable target in joint disease.

In conclusion, IFP-MSC exhibit enhanced stemness potential, comparable MSC-related phenotypic profile, superior chondrogenic capacity and acquisition of a strong immunomodulatory phenotypic, secretory and functional cell signature after priming with inflammatory and pro-fibrotic conditions, compared to BM-MSC. Although performed in a limited number of donors for IFP- and BM-MSC (n = 3 each), these results help elucidate various local activities of IFP-MSC within the IFP during joint disease pathogenesis and progression. In parallel, they help to shed light on the benefits of priming the cell-based product during the manufacturing process before cell therapy for OA and potentially to other inflammation/fibrosis-related entities such as Rheumatoid Arthritis (RA) where the IFP shows key roles^75,76^. From a therapeutic perspective, the local activities of injected MSC may be related not only with control of immune-related responses, as tested here, but potentially also with pain control (ongoing research) as SP has been described as the main nociceptive neurotransmitter within joints given its production by sensory nerves. The results obtained here offer valuable information establishing similarities and differences between MSC from different sources in terms of their responsiveness, potency and functional displays. Furthermore, they conceptually support the notion of cell priming with specific cocktails (*e*.*g*., addition of CTGF to inflammatory inductors) prior to the therapy aiming at inducing/enhancing specific desired traits and attributes, rendering the manufactured cell-based product more potent and/or the results of the therapy potentially more reproducible depending on the clinical stage.

## Methods

### Isolation, Culture and Expansion of IFP-MSC and BM-MSC

Following informed consent, IFP-MSC were isolated from IFP tissue obtained from de-identified, non-arthritic patients (two males and one female, 46, 56 and 59 years-old, respectively) determined by MRI, undergoing elective knee arthroscopy for anterior cruciate ligament (ACL) reconstruction at the Lennar Foundation Medical Center - University of Miami. All procedures were carried out in accordance with relevant guidelines and regulations and following an approved protocol by the University of Miami Institutional Review Board (IRB), which determined it not as human research based on the nature of the samples as discarded tissue. Harvested healthy IFP tissue (<20 ml) was mechanically dissected and washed repeatedly with Dulbecco’s Phosphate Buffered Saline (DPBS; Sigma), followed by enzymatic digestion using 235 U/ml Collagenase I (Worthington Industries, Columbus, OH) diluted in DPBS and 1% bovine serum albumin (Sigma) for 2 hours at 37 °C with agitation. Cell digests were inactivated with complete media [DMEM low glucose + GlutaMAX (ThermoFisher Scientific, Waltham, MA) + 10% fetal bovine serum (FBS; VWR, Radnor, PA], washed and seeded at a density of 1 × 10^6^ cells/175 cm^2^ flask in complete media.

BM-MSC were isolated from BM aspirates obtained from de-identified healthy donors (two females and one male, 30, 49 and 48 years-old, respectively) after provided written informed consent. All procedures were carried out in accordance with relevant guidelines and regulations and following a protocol approved by the Case Stem Cell Facility IRB (approval # 09-90-195). Mononuclear cells (MNCs) obtained from BM aspirates using Percoll (Sigma, St. Louis, MO) were seeded at a density of 0.2 × 10^6^ cells/175 cm^2^ flask in complete media (DMEM low glucose + GlutaMAX + 10% foetal bovine serum).

Both MSC were cultured at 37 °C 5% (v/v) CO_2_ until 80% confluent (denoted as P0), then passaged at a 1:10 ratio until P3 detaching them with TrypLE™ Select Enzyme 1X (Gibco, ThermoFisher Scientific) and assessing cell viability with 0.4% (w/v) Trypan Blue (Invitrogen, Carlsbad, CA).

### Clonogenic assay

Both naïve P3 MSC were seeded in 100 mm culture plates in duplicate at a density of 10^3^ cells/plate in complete medium. On day 10, colony-forming unit fibroblasts (CFU-Fs) were manually enumerated after cytochemical staining with 0.01% Crystal Violet (Sigma).

### MSC priming

Both naïve P3 MSC (n = 3) were primed with TI inflammatory mediators (15 ng/ml TNFα, 10 ng/ml IFNγ) for 48 h or TIC inflammatory/fibrotic mediators (15 ng/ml TNFα, 10 ng/ml IFNγ, 10 ng/ml CTGF) for 72 h. The time difference is based on an attempt to mimic the cascade of events that lead from “pure” inflammation (TI) to a more fibrotic response (TIC), as a sequential tissue response (synovitis followed by IFP fibrosis).

### Cell growth kinetics measurement

P3 naïve and primed MSC were seeded in 6-well plates at a density of 10^4^ cells/well in complete medium. Growth curves were generated from bright field image obtained using IncuCyte® Live Cell Analysis System with IncuCyte ZOOM® software (Essen Bioscience, Ann Arbor, MI) to quantify cell confluence as a percentage for a 10-day period.

### Immunophenotype

Flow cytometric analysis was performed on P3 naïve and primed IFP (n = 3) and BM (n = 3) MSC. 2.0 × 10^5^ cells were labelled with monoclonal antibodies specific for: CD10, CD44, CD56, CD73, CD90, CD105 (Biolegend, San Diego, CA), CD146, LepR (Miltenyi Biotec, Auburn, CA), CD166, CD271, NG2 (BD Biosciences, San Jose, CA), CD200, CXCR4 (Invitrogen) and the corresponding isotype controls (Supplementary Table S2). All samples included a Ghost Red Viability Dye (Tonbo Biosciences, San Diego, CA). Data were acquired using a Cytoflex S (Beckman Coulter, Brea, CA) and analysed using Kaluza analysis software (Beckman Coulter).

### Quantitative real-time PCR (qPCR)

RNA extraction was performed in both MSC types (P3, n = 3 each) before (naïve) and after TI/TIC exposure (primed), using the RNeasy Mini Kit (Qiagen, Frederick, MD) according to manufacturer’s instructions. Total RNA (1 μg) was used for reverse transcription with SuperScript™ VILO™ cDNA synthesis kit (Invitrogen), and 10 ng of the resulting cDNA was analysed by qPCR using QuantiFast SYBR Green qPCR kit (Qiagen) and a StepOne Real-time thermocycler (Applied Biosystems, Foster City, CA). For each target, human transcript primers were selected using PrimerQuest (Integrated DNA Technologies, San Jose, CA) (Supplementary Table S3). All samples were analysed in triplicate. Mean values were normalized to GAPDH, expression levels were calculated using the 2^−ΔΔ^Ct method and represented as the relative fold change of the primed cohort to the naïve (=1).

### Chondrogenic differentiation

Chondro-pellet cultures (0.25 × 10^6^ MSC) were induced towards chondrogenesis for 21 days with serum-free MesenCult-ACF differentiation medium (STEMCELL Technologies Inc, Vancouver, Canada). Harvested pellets were cryosectioned and 6-μm frozen sections stained with 1% toluidine blue (Sigma). Sulfated glycosaminoglycans (sGAG) were quantified after pellet digestion at 65 °C overnight with 1 mg/ml papain (Sigma) solution using the Blyscan Glycosaminoglycan Assay (Biocolor, Carrickfergus, UK) according to manufacturer’s instructions. DNA content was quantified using Fluorescent DNA Quantitation Kit (Bio-Rad Laboratories, Hercules, CA) according to manufacturer’s instructions.

### Secretome analysis

Arrays for growth factors (GFs) and inflammatory mediators (RayBio^®^ C-Series, RayBiotech, Peachtree Corners, GA) were used to determine secreted levels obtained from culture-expanded IFP and BM-MSC pre- and post-priming. For each population, 1 mL of conditioned media obtained from 2 donors, was prepared and used for each assay following the manufacturer’s instructions. Data shown represent 40 sec exposure in FluorChem E chemiluminescence imaging system (ProteinSimple, San Jose, CA). Results were generated by quantifying the mean spot pixel density of each array using protein array analyser plugin using ImageJ software (Fiji/ImageJ, NIH website). The signal intensities were normalized with the background whereas separate signal intensity results represent the average pixel density of two spots per protein. The signal intensity for each protein spot is proportional to the relative concentration of the antigen in the sample.

### Pathway analysis

Putative interactomes were generated by Search Tool for Retrieval of Interacting Genes/Proteins (STRING 11.0; available from: http://string-db.org)^77^ database using interaction data from experiments, databases, neighbourhood in genome, gene fusions, co-occurrence across genomes, co-expression and text-mining. An interaction confidence score of 0.4 was imposed to ensure high interaction probability. K-means clustering algorithm was used to organize proteins into 3 separate clusters per condition tested, discriminated by colours. Venn diagram was used to demonstrate all possible relations between IFP- and BM-MSC post-TI and -TIC priming for the significantly (*p < 0*.*05*) altered proteins. Functional enrichments related to biological process, Kyoto Encyclopedia of Genes and Genomes (KEGG) pathways, and reactome pathways were presented in radar graphs for all conditions tested.

### Substance P *in vitro* assay

Parameter Substance P competitive immunoassay (R&D Systems, MN) was used to quantify the levels (in pg/ml) of endogenous and exogenously-added SP to culture-expanded MSC (10^5^/well, 12-well; n = 3 for each MSC type) before and after TI and TIC priming, following manufacturer’s instructions. After 24 hours, cells were switched to TI or TIC priming media for 48 and 72 hours, respectively. SP was then quantified in centrifuged (1500 rpm; 5 minutes) conditioned media (in technical triplicates run in duplicates within the membrane) obtained from naïve-, TI- and TIC-induced MSC: i) in baseline cultures (*i*.*e*., endogenous MSC-derived SP); ii) after exogenous addition of substance P (624 pg/ml) for 35 minutes to the cell-free supernatant (*i*.*e*., supernatant group); and iii) after addition of SP (624 pg/ml) for 35 minutes to the cells in fresh medium (*i*.*e*., cells group). Parallel wells of supernatants and cells were treated with the CD10 inhibitor thiorphan (5 µg/ml) 30 minutes before and during SP addition. SP final levels were determined by subtracting measured optical densities of individual wells at 450 nm and 540 nm (SpectraMax M5 spectrophotometer, Molecular Devices, San Jose, CA), and converted into concentrations using the reference standard curve run with the assay, and contrasted to samples with only exogenously-added SP to the medium (*i*.*e*., no cells and no supernatant).

### CD10 immunolocalization

Both MSC groups were fixed with 4% paraformaldehyde, washed with PBS, followed by incubation with tris buffered saline (TBS; Sigma-Aldrich) containing 0.05% Triton X-100 solution (Sigma-Aldrich) for 30 minutes. Groups were then incubated with blocking solution composed of TBS with 10% normal goat serum (NGS) for 1 hour. Goat anti-human CD10 polyclonal antibody (R&D Systems, Supplementary Table S2) was prepared in TBS containing 1% NGS and added to samples for 1 hour incubation at room temperature with gentle agitation. Samples were washed with TBS and incubated for 1 hour with secondary antibody containing AlexaFluor594 conjugated rabbit anti-goat IgG antibody combined with DAPI (Thermo Fisher Scientific) at room temperature with gentle agitation in the dark. TBS was used to wash cells, and microscope images were acquired using Leica DMi8 microscope with Leica X software (Leica).

### Mono-iodoacetate model of acute synovial/IFP inflammation

The animal protocol was approved by the Institutional Animal Care and Use Committee (IACUC) of the University of Miami, USA (approval no. 16-008-ad03) and conducted in accordance to the ARRIVE guidelines^78^. Sixteen (#16) 10-week old Sprague Dawley rats (8 males and 8 females; mean weight 250 g and 200 g, respectively) were used. The animals were housed to acclimate for 1 week before the experiment initiation. One rat was housed per cage in a sanitary, ventilated room with controlled temperature, humidity, and under a 12/12 hour light/dark cycle with food and water provided ad libitum.

Acute synovial/IFP inflammation was generated by intra-articular injection of 1 mg of MIA in 50 µl of saline in the right knee. This short exposure to MIA has been shown to induce inflammatory changes at the synovium and adjacent IFP^70^. Briefly, under isoflurane inhalation anesthesia rat knees were flexed 90° and MIA was injected into the medial side of the joint with a 27 G needle using the patellar ligament and articular line as anatomical references. Three (3) days later, a single intra-articular injection of 100,000 IFP-MSC in 50 µl of Euro-Collins solution (MediaTech) was performed also in the right knee of 12 rats (similar injection technique). Animals were sacrificed at two different timepoints: 3 and 7 days after IFP-MSC injection (d6 and d10 total, respectively, n = 6 each). As controls, 4 rats received MIA in the right knee (Only MIA group), and only IFP-MSC in the left knee (Only IFP-MSC group).

### Substance P and Human Mitochondria immunolocalization

Rat knee joints were harvested by cutting the femur and tibia/fibula 1 cm above and below the joint line, muscles were removed and joints were fixed with 10% neutral buffered formalin (Sigma-Aldrich) for 14 days at room temperature. Knee joints were decalcified, cut at sagittal plane in half, embedded in paraffin and serial 4 μm sections were obtained. Hematoxylin and Eosin (H&E) staining was performed to evaluate the structure and morphology of knee joints. Substance P and anti-human Mitochondria immunolocalization were determined by immunofluorescence and immunohistochemistry staining, respectively.

For anti-substance P immunofluorescence staining, sections were incubated with 1x citrate buffer solution at 60 °C overnight for antigen retrieval, permeabilised with 1× PBS + 0.2% Triton X-100 for 20 minutes at room temperature, and incubated with blocking buffer (1× PBS + 0.1% Triton X-100 with 10% rabbit serum) for 1 hour at room temperature. In between different treatments sections were washed with 1× PBS. Rabbit anti-rat substance P polyclonal antibody (Millipore, Supplementary Table S2) was prepared in blocking buffer (1:100) and sections were incubated at 4 °C overnight. Sections were washed with 1× PBS + 0.01% Triton X-100 and incubated for 1 hour with secondary antibody containing Alexa Fluor594 conjugated goat anti-rabbit IgG antibody (Thermo Fisher Scientific) at room temperature. Controls were incubated with secondary antibody only. All sections were rinsed with 1× PBS, mounted in prolong gold antifade reagent with DAPI (Invitrogen), and microscope images were acquired using Leica DMi8 microscope with Leica X software (Leica).

For anti-human mitochondria immunohistochemistry staining, sections were treated using the mouse specific HRP/DAB (ABC) detection IHC kit (abcam) according to the manufacturer’s instructions. Briefly, HRP-labelled streptavidin mouse anti-human mitochondria monoclonal antibody (Millipore, Supplementary Table S2) was prepared in 1× PBS containing 1% bovine serum albumin (1:150) and sections were incubated for 2 hours at room temperature. In between different treatments sections were washed with TBS and then incubated with biotinylated anti-mouse secondary antibody. Controls were incubated with secondary antibody only. At the end of the immunohistochemistry staining all sections were mounted in cytoseal xyl (Thermo scientific), and microscope images were acquired using Leica DMi8 microscope with Leica X software (Leica).

### Immunopotency assay (IPA)

IFP-MSC were seeded in 24 well plates at a density of 1.5 × 10^5^, 7.5 × 10^4^, 2.5 × 10^4^, 5 × 10^3^ or 0 MSC/well. Cells were cultured for 48 hours in MSC media alone (naïve) or TI-supplemented. After 48 hours, 3.0 × 10^5^ CFSE-labelled human PBMC, obtained from de-identified donors after provided written informed consent, performed in accordance with relevant guidelines and regulations and following a protocol approved by the University of Miami IRB (approval #19950119), were added to each well in MLR culture media (RPMI 1640 + GlutaMAX (Invitrogen) supplemented with 15% heat inactivated normal human AB serum (Corning, Corning, NY), 1% penicillin-streptomycin, 1% non-essential amino acids, 1% sodium pyruvate, 1% vitamins (Invitrogen) and 2 mM HEPES (Corning Mediatech, NY). PMA/IO 500× (ThermoFisher Scientific) was added to stimulate PBMC proliferation, with unstimulated cells used as controls. Cultures were incubated at 37 °C, 5% CO_2_ for 4 days, harvested, stained with the viability dye, Live/Dead (ThermoFisher Scientific), and analysed for CFSE + proliferation on a BD LSR II flow cytometer.

### Statistical analysis

Normal distribution of values was assessed by the Kolmogorov-Smirnov normality test. Statistical analysis was performed using paired and unpaired Student’s *t*-test for normally distributed data and Wilcoxon (for paired data) or Mann Whitney (for unpaired data) test in presence of a non-normal distribution; one-way ANOVA was used for multiple comparisons. All tests were performed with GraphPad Prism v7.03 (GraphPad Software, San Diego, CA). Level of significance was set at *p* < 0.05. Data used for the statistical analyses is indicated in the figure legends, overall corresponding to three independent experiments from different MSC donors (n = 3), unless specified. The immunopotency assay has more data points as each MSC donor was tested with two different PBMC populations also obtained from independent donors.

## Supplementary information

Supplementary Information

## Data Availability

All data generated or analysed during this study are included in this published article (and its Supplementary Information files).
