# Supplementary material for: Infrapatellar fat pad-derived MSC response to inflammation and fibrosis induces an immunomodulatory phenotype involving CD10-mediated Substance P degradation
Source: Sci Rep. 2019 Jul 26;9:10864. doi: 10.1038/s41598-019-47391-2 (PMC6659713; doi:10.1038/s41598-019-47391-2)
Supplement: Supplementary file 1 — Supplementary Information [file 41598_2019_47391_MOESM1_ESM.pdf]

Infrapatellar fat pad-derived MSC response to inflammation and fibrosis induces an immunomodulatory phenotype involving CD10-mediated Substance P degradation

Dimitrios Kouroupis,[1,2] Annie Bowles,[1,2,3] Melissa Willman,[2] Carlotta Perucca Orfei,[4] Alessandra Colombini,[4] Thomas Best,[1] Lee Kaplan,[1] and Diego Correa,*[1,2]

Supplementary Table S1: Selected pathways and proteins involved post-TI and TIC priming. Related to Figure 4.

IFP-MSC naïve vs TI

PATHWAYS	PROTEINS
MAPK signaling	FLT4 IGF1R TGFA FIGF IGF1 AREG NTF3 TNF NTF4
PI3K-Akt signaling	FLT4 IGF1R TGFA FIGF IGF1 IL6R AREG NTF3 NTF4
Ras signaling	FLT4 IGF1R TGFA FIGF IGF1 NTF3 NTF4
IL-10 signaling	ICAM1 CXCL10 CXCL8 TNFRSF1B TNF CCL5
IL-4 and IL-13 signaling	ICAM1 CXCL8 IL6R TNFRSF1B TNF
TNF signaling	ICAM1 CXCL10 TNFRSF1B TNF CCL5
Rheumatoid arthritis	IFNG ICAM1 CXCL8 TNF CCL5

IFP-MSC naïve vs TIC

PATHWAYS	PROTEINS
MAPK signaling	FGF4 FGF2 EGF IGF1R CSF1 NGF AREG TNF
IL-10 signaling	ICAM1 CSF2 CXCL10 CXCL8 CSF1 TNFRSF1B TNF CCL5
TNF signaling	ICAM1 CSF2 CXCL10 CSF1 TNFRSF1B TNF CCL5
PI3K-Akt signaling	FGF4 FGF2 EGF IGF1R CSF1 NGF AREG
Rheumatoid arthritis	IFNG ICAM1 CSF2 CXCL8 CSF1 TNF CCL5
Ras signaling	FGF4 FGF2 EGF IGF1R CSF1 NGF
IL-4 and IL-13 signaling	FGF2 ICAM1 CXCL8 TNFRSF1B TNF

BM-MSC naïve vs TI

PATHWAYS	PROTEINS
MAPK signaling	HGF EGFR PDGFA TGFB2 AREG TNF NTF4
PI3K-Akt signaling	HGF CSF3 EGFR PDGFA AREG NTF4
IL-10 signaling	CSF3 ICAM1 CXCL10 TNF CCL5
Rheumatoid arthritis	IFNG ICAM1 TGFB2 TNF CCL5
TNF signaling	ICAM1 CXCL10 TNF CCL5

BM-MSC naïve vs TIC

PATHWAYS	PROTEINS
Regulation of actin cytoskeleton	FGF4 FGF6 PDGFRA PDGFRB FGF2 EGF EGFR PDGFB PDGFA
IL-10 signaling	CSF3 CCL2 ICAM1 CSF2 CXCL10 CSF1 IL6 TNF CCL5
TNF signaling	CCL2 ICAM1 CSF2 CXCL10 CSF1 IL6 TNF CCL5

Supplementary Table S2. List of Antibodies. Related to Figures 2, 5, and 6.

ANTIBODY	CLONE	CONCENTRATION	CAT#	MANUFACTURER
CD10-APC	HI10a	5ul undiluted	312210	Biolegend
CD44-BV605	IM7	5ul undiluted	103047	Biolegend
CD56-FITC	HCD56	5ul undiluted	318304	Biolegend
CD73-APC	AD2	5ul undiluted	344006	Biolegend
CD90-FITC	5E10	5ul undiluted	328108	Biolegend
CD105-PE	SN6h	5ul undiluted	800504	Biolegend
CD146-PE	541-10B2	5ul undiluted	130-092-853	Miltenyi Biotec
CD166-BV786	3A6	5ul undiluted	564939	BD Biosciences
CD200-FITC	OX104	5ul undiluted	11-9200-42	Invitrogen
CD271-PECF594	C40-1457	5ul undiluted	563452	BD Biosciences
NG2-AlexaFluor647	9.2.27	5ul undiluted	562414	BD Biosciences
LepR-PE-Vio770	REA361	5ul undiluted	130-105-153	Miltenyi Biotec
CXCR4-APC	12G5	5ul undiluted	17-9999-42	Invitrogen
Anti-CD10	Polyclonal	1:20 diluted	AF1182	R & D Systems
Anti-Substance P	Polyclonal	1:100 diluted	AB1566	Millipore
Anti-mitochondria	113-1	1:150 diluted	MAB1273	Millipore

Supplementary Table S3. List of transcripts and primers. Related to Figure 3.

TRANSCRIPT NAME	PRIMERS
IDO	Forward: *AGA GTC AAA TCC CTC AGT CC*<br>Reverse: *AAA TCA GTG CCT CCA GTT CC*
IL-6	Forward: *GTA GCC GCC CCA CAC AGA CAG CC*<br>Reverse: *GCC ATC TTT GGA AGG TTC*
IL-8	Forward: *GAA CTG AGA GTG ATT GAG AGT*<br>Reverse: *CTT CTC CAC AAC CCT CTG*
CD10	Forward: *CTG TGG GAT GAG GAG GTT AAA G*<br>Reverse: *GGA GGC TAA AGC AGG AGA ATA G*
ICAM-1	Forward: *AGG AGG TGG TAA GAG AGA AGA G*<br>Reverse: *TAA GGG TGG GAG GAG GAT TT*
G-CSF	Forward: *AGC TTC CTG CTC AAG TGC*<br>Reverse: *TTC TTC CAT CTG CTG CCA GAT GGT*
HGF	Forward: *ATG TGG GTG ACC AAA CTC CTG*<br>Reverse: *CTA TTG AAG GGG AAC CAG AGG*
IGF-1	Forward: *TCT TGA AGG TGA AGA TGC ACA CCA*<br>Reverse: *AGC GAG CTG ACT TGG CAG GCT TGA*
IL-10	Forward: *GCC TAA CAT GCT TCG AGA TC*<br>Reverse: *TGA TGT CTG GGT CTT GGT TC*
TGFβ	Forward: *CAG CAA CAA TTC CTG GCG ATA*<br>Reverse: *AAG GCG AAA GCC CTC AAT TT*
HLA-G	Forward: *GCT GCT GTG CTG TGG AGA A*<br>Reverse: *TCT GGA ACA GGA AAG GTG ATT GG*
PTGES2	Forward: *CCT GGA AGA GAT CAT CAC C*<br>Reverse: *CCT TCT CGT TGA GCA TGA*
GAPDH	Forward: *TAC GTC GTG GAG TCC ACT GG*<br>Reverse: *GCC AAC GTG TCA GTG GTG GA*
